# Integrating alternative splicing detection into gene prediction

**DOI:** 10.1186/1471-2105-6-25

**Published:** 2005-02-10

**Authors:** Sylvain Foissac, Thomas Schiex

**Affiliations:** 1 Unité de Biométrie et Intelligence Artificielle, INRA, 31326 Castanet Tolosan, France

## Abstract

**Background:**

Alternative splicing (AS) is now considered as a major actor in transcriptome/proteome diversity and it cannot be neglected in the annotation process of a new genome. Despite considerable progresses in term of accuracy in computational gene prediction, the ability to reliably predict AS variants when there is local experimental evidence of it remains an open challenge for gene finders.

**Results:**

We have used a new integrative approach that allows to incorporate AS detection into *ab initio *gene prediction. This method relies on the analysis of genomically aligned transcript sequences (ESTs and/or cDNAs), and has been implemented in the dynamic programming algorithm of the graph-based gene finder EuGÈNE. Given a genomic sequence and a set of aligned transcripts, this new version identifies the set of transcripts carrying evidence of alternative splicing events, and provides, in addition to the classical optimal gene prediction, alternative optimal predictions (among those which are consistent with the AS events detected). This allows for multiple annotations of a single gene in a way such that each predicted variant is supported by a transcript evidence (but not necessarily with a full-length coverage).

**Conclusions:**

This automatic combination of experimental data analysis and *ab initio *gene finding offers an ideal integration of alternatively spliced gene prediction inside a single annotation pipeline.

## Background

Alternative splicing (AS) is a biological process that occurs during the maturation step of a pre-mRNA, allowing the production of different mature mRNA variants from a unique transcription unit. AS is known to play a key role in the regulation of gene expression and transcriptome/proteome diversity [1]. First considered as an exceptional event, AS is now thought to involve the majority of the human multi-exon genes, from 50% to 74% [1-3]. This observation raises new issues for genome annotation, especially concerning the computational gene finding process that generally provides only one exon-intron structure per sequence.

In the context of structural gene prediction, two classes of approaches are usually considered. In the first approach, usually denoted as *intrinsic *or *ab initio*, the only type of information used for gene prediction lies in the statistical properties of the various gene elements (exons, splice sites and other biological signals). On the contrary, so-called *extrinsic *approaches essentially rely on the existence of similarities between the sequence to annotate and other known sequences (either proteins, transcripts or other genomic sequences). Several existing gene finding tools are essentially *intrinsic *(or *ab initio*): this is the case for Genscan [4], HMMgene [5] or SLAM [6]. For such a gene finder, the predicted gene structure is defined as an optimal prediction, that is the most probable according to its underlying probabilistic model. In the presence of AS however, a unique prediction is not sufficient. One obvious possibility is to look for suboptimal predictions. This can be done for a classic HMM-based gene finder by a modification of the Viterbi algorithm, thus providing the set of the *k *best predictions. This approach has been applied *eg*. in HMMgene or in FGENES-M (unpub.). Another way to obtain suboptimal solutions from a HMM is to do HMM sampling [7]. This method, which consists in randomly generating parses according to the posterior probabilities, has been implemented in the gene finder SLAM. Usually, a very large amount of samples are needed to generate just a single prediction that differs from the optimal one. Genscan adopt a different approach and search for alternative exons not represented in the optimal prediction. This is done using a forward-backward algorithm to identify potential exons for which the *a posteriori *likelihood is larger than a given threshold.

In addition to the fact that all these exclusively *intrinsic *approaches cannot take into account transcript evidences, they suffer from two major problems of sensibility and specificity:

First of all, these methods assume that predictions representing AS variants should have a probability which is very close to the optimal probability according to the underlying gene model. This is however quite arguable, especially when the alternative structure significantly differs from the optimal one. Actually, when an AS variant *eg*. shifts from a strong to a weak or a non-consensus splice site or shows a complete coding exon skipping event, it is quite unlikely that the probability will remain in the neighborhood of the optimum since it will not be able to incorporate the corresponding splicing or coding score.

Moreover, a strong specificity problem has been observed for this approach. Since a very large number of alternative predictions can always be produced for any sequence, it is essential to be able to distinguish those reflecting real AS variants from *in silico *false positives. To perform this, and as long as AS sites dedicated prediction tools are unavailable, the probability of a prediction alone cannot be sufficient and additional evidence is required.

In opposition to the purely *intrinsic *approach, the analysis of experimental data can provide useful information. More specifically, sequences of mature transcripts resulting from AS provide reliable evidence of the existence of the AS event. Large scale studies have already been undertaken to detect AS evidences from transcript alignments and to collect them in databases such as *eg*. HASDB [8], ASDB [9], ASAP [10], ASD [11], EASED [12] or ProSplicer [13]. Some software tools have also been designed to perform and/or exploit transcript alignment with the aim of identifying alternative gene structures. Such *extrinsic *annotation tools include GeneSeqer [14], ASPic [15], TAP [16,17], and PASA [18]. Except for GeneSeqer which is more focused on performing spliced alignment, the three other software adopt the same strategy: using genomically aligned transcripts, the aim is to determine the exon-intron structure(s) compatible with the greatest number of transcripts. Another approach, Cluster Merge [19], has been recently used in the Ensembl annotation system [20] to identify minimal sets of transcript variants compatible with genomically aligned ESTs evidences.

Unlike *intrinsic *methods, *extrinsic *approaches take advantage of transcript information. However, they also suffer from some limitations : first they entirely depend on the availability of transcribed sequences which bounds their sensitivity. With little exceptions (like TAP that exploits genomic sequence properties to identify gene boundaries, including *eg*. a polyA site scanning step, or GeneSeqer, that contains an *intrinsic *splice sites scoring method), they cannot predict a splice site if it is not represented in a transcript-to-genome alignment and therefore require a total coverage of each gene with all exon-intron boundaries. This can be problematic considering the ESTs fragmented nature. Moreover, when such methods can take advantage of a total gene coverage, the CDS localization remains to be done and the pure transcript predictions may not respect elementary coding gene properties (such as the presence of an ORF w.r.t. a given frame). Furthermore, overlapping transcripts are sometimes assumed to come from the same mature mRNA and are therefore merged. This may lead to the fusion of two overlapping transcripts coming from exclusive inconsistent mRNA variants, thus forcing the prediction to respect a chimeric virtual assembly.

Finally, and because experimental transcripts cannot exist for every existing gene, both *intrinsic *and *extrinsic *information are needed inside an annotation pipeline [20]. The predictions provided by two different approaches can be different and even inconsistent, and merging them together requires a careful inspection of human curators, as performed in [18]. A fully integrative method alleviates all these problems. GrailEXP [21] seems to be the only gene finder that tried to go in this direction. However, it can only consider AS events leading to complete exon inclusion/retention, ignoring thus approximatively half of the AS cases [8,18]. The underlying approach remains unpublished.

To extend the domain of application of gene prediction to alternatively spliced gene structure prediction, we have designed an *intrinsic/extrinsic *integrative annotation method with the following aims:

• For a given genomic sequence, an optimal gene structure prediction is produced, as usual.

• In addition to this optimal prediction, for every transcript sequence providing evidence of AS, an optimal prediction consistent with this splicing form is also provided.

• Each additional or alternative gene structure prediction has to be supported by some biological evidence.

• Full-length transcript coverage is not required for a complete gene structure identification.

• Each prediction satisfies the usual constraints on gene structure. A correct proteic coding gene is defined by a succession of one or more exons separated by introns flanked by splice sites. It contains a CDS between a start and a stop codon, and no in-frame stop in coding exons.

Our aim is to combine the advantages of the *intrinsic *and *extrinsic *approaches in an integrative system allowing for AS detection based on the analysis of genomic aligned transcript sequences. The method has been implemented inside EuGÈNE-M, a new version of the *Arabidopsis thaliana *EuGÈNE gene finder [22,23], and applied to a reference genes set.

## Results

**Figure 1:**
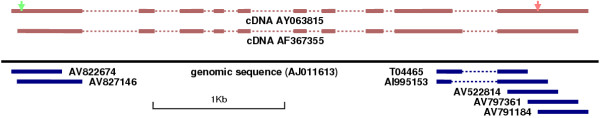
**EST/cDNA alignments on the spl7 gene region**. Thick lines represent matches an dotted lines, gaps. Above the genomic sequence, the 2 full-length cDNAs that provide the two correct reference gene structures are presented. Arrows indicate the start and stop codons. The ESTs T04465 and AI995153 present inconsistent splicing profiles and are labeled as *incompatible*.

To evaluate the interest of EuGÈNE-M compared to existing transcripts-based approaches, we applied it on the *spl7 Arabidopsis thaliana *gene. This gene codes for the squamosa promoter-binding protein-like 7, has 10 exons and two known alternative mature mRNA variants, both supported by a distinct full-length cDNA (accession AY063815 and AF367355, Figure 1). The genomic alignments of these cDNAs provide two correct and reliable gene structures used as reference annotations. The structures differ only by the 3' extremity of the 9th exon. However, beyond these 2 complete cDNAs, only the first and the two last exons are covered by ESTs. This partial EST coverage configuration is interesting because without the full-length cDNAs (unavailable in dbEST), finding a correct gene structure with pure *extrinsic *assembly tools would not be possible.

Given only the genomically ESTs alignments, we applied EuGÈNE-M on the genomic sequence containing the *spl7 *gene. Since two ESTs (T04465 and AI995153) show *incompatible *alignments (see Methods), EuGÈNE-M computes two additional predictions, each being consistent with one of them. The first *alternative prediction *is the same as the optimal one and corresponds to one variant; the second corresponds to the other variant.

For a more extensive test, we applied EuGÈNE-M on AraSet [24], a data set of *Arabidopsis thaliana *curated genes recently used in the assessment of GeneSeqer [25]. Since EuGÈNE has already been evaluated on this benchmark set, performing as one of the most accurate gene finder [22], the aim of this test is to provide an estimation of an alternatively spliced genes ratio on a reference set. Predictions are available in the additional files. On the 168 AraSet reference genes, 9 show at least two *alternative predictions*, that were carefully analyzed. This is summarized in Table 1. All these predictions but two correspond to potential alternative splicing events. Among the two remaining ones, a first predicted AS event corresponds to an incompatibility caused by an apparently incompletely spliced EST. The other is more interesting since it is caused by two ESTs from two different genes lying on opposite strands and overlapping on their 3' ends. In this case, EuGÈNE-M is forced to predict two overlapping genes, one on each strand, which effectively address the usual impossibility for existing gene models to predict overlapping genes. Of course, these predictions, as all *in silico *expertise, require experimental verifications to be confirmed. If we assume the 7 remaining genes are effectively subject to AS, this yield to an AS rate of ~4.2%, a ratio in the same order as previously estimated, from 1.5% [26] to 6.5% (computed from [18]).

## Discussion

In the recent assessment of GeneSeqer on this AraSet data set, only three AS cases were reported [25]. However, the authors only reported AS cases that were detected in GeneSeqer high-quality alignments and producing introns differing from the AraSet annotated introns. We therefore verified that our alignments were consistent with the GeneSeqer assessment alignment data available in the *Arabidopsis thaliana *Genome Database AtGDB [27,28]. We noticed an alignment difference for only one of our alternative EST (CF652136), not present in the AtGDB because of its dbEST entry date (Oct. 2003). We also checked if the AS variants predicted by EuGÈNE-M were already reported in the AS sections of the AtGDB [26,29] and of the TIGRdb [18,30]. Only 3 of our detected AS predictions were already reported in both databases, and 3 were missing in all of them (Table 1), confirming that this methodology can help to automatically discover new potential AS cases, even on a well studied dataset.

The analysis of these AS cases confirms that AS seems to be much less frequent in *A. thaliana *than in *Homo sapiens*. Nevertheless, this AS ratio estimation is expected to increase in the future with the growth of transcript data availability. Another interesting point is the nature of the variants: on this gene set, the majority of AS cases involves a simple acceptor or donor alternative splice site. Notice however that since EuGÈNE-M's underlying model allows arbitrary alternative gene structure to be predicted, it is not limited to the prediction of such simple AS events and can perfectly cope with complex AS events, as found in mammals. This methodology can also be integrated in other existing gene finders where the score of a gene structure is defined as the sum of elementary scores of the signals and nucleotides involved in the gene structure (this includes HMM-based gene finders).

## Conclusions

In this paper we have presented a new method to deal with alternative splicing in annotation and gene prediction. This integrative approach combines the advantages of an *intrinsic *and an *extrinsic *process to incorporate AS detection into *ab initio *gene finding. We showed that this method allows the discovery of new alternative spliced genes, with the reliability of *extrinsic *annotation and the potential exhaustiveness of *ab initio *gene prediction.

## Methods

The process that goes from the original genomic sequence and associated aligned transcripts to the AS prediction is composed of three steps which we rapidly describe here :

• first, the set of genomically aligned transcripts is analysed to detect AS evidences on the basis of splicing inconsistency between transcripts variants.

• Then, the graph-model used in EuGÈNE to model potential gene structures is modified to take into account these aligned transcripts. For each transcript variant, the graph used in EuGÈNE for gene structure prediction is connected to an additional parallel graph subunit where local constraints are injected according to the exon-intron information provided by the corresponding transcript alignment.

• Finally, an extended version of the dynamic programming algorithm used for obtaining an optimal prediction allows to identify, for each graph subunit, the best prediction consistent with the corresponding transcript alignment.

### Detection of AS evidences from transcripts analysis

Since EuGÈNE already exploits transcripts information to improve the gene prediction process [22], the AS prediction only requires to consider transcripts providing evidence of AS. With this purpose, we focus on inconsistencies between transcript alignments.

Transcript sequences are first aligned against the genomic sequence using a spliced alignment tool. The choice of the source transcript database and the alignment tool is not *a priori *imposed by the method. Transcript sequences in our analysis were extracted from the *A. thaliana *section of dbEST [31] (release Dec. 2003: 190, 708 entries), and aligned in two steps. For the first step we used sim4 [32], a fast software that can deal with huge EST datasets. In the second step, we used GeneSeqer [14], usually more accurate on splice junction identification, to realign all transcripts aligned by sim4 that passed the following filtering process.

A first filtering step is performed on the basis of the transcript sequence and alignment quality. To be considered, an alignment has to satisfy some constraints defined by filtering parameters. For *Arabidopsis thaliana*, default parameters values are set as following: transcript length between 30 and 10000 bp, minimum alignment length = 95% of the transcript length, minimum identity score of 97%, maximum gap length of 5000 bp, maximum match length of 4000 bp. By default, and to avoid genomic contamination, unspliced transcripts are removed from the analysis. Moreover, because of the frequent weak alignment quality at the terminal regions, alignments extremities are shortened (by 15 bp by default).

The second filtering step depends on the relation between transcript alignments. To detect AS evidences, every pair of overlapping transcript alignments is analyzed. We consider two special types of pairwise relation : a transcript alignment *A *is labeled as *included *in *B *if and only if for each genomic position of *A *the same genomic position in *B *shares the same alignment information (either gap or match). Every transcript *included *in another transcript is ignored by default. Transcript alignments *A *and *B *are labeled as *incompatible *if and only if there is a genomic position for which both ESTs are informative and give an inconsistent information, that is a gap (representative of the presence of an intron) is faced with a match (representative of the presence of an exon, coding or not). Examples of *incompatible *ESTs are displayed in Figure 1 and 5. Since we focus on AS evidences, we only keep transcripts labeled as *incompatible *after all pairwise comparisons. Considering orientation of ESTs, the information on the clone-sequencing orientation that can be found with ESTs is totally ignored in this filtering process because of its unreliability. In practice, spliced EST can be reliably oriented by looking for splice sites on the hit-match frontier of the EST alignments and by choosing the strand for which such splice sites exist. The parameters of these two automatic filtering steps can be modified by the user through a simple text file. We will denote the resulting transcript alignments kept as *alternative transcripts*.

### The gene-finder EuGène

#### General description

EuGÈNE is a gene finding software based on a directed acyclic graph gene model [22]. For each nucleotide of the genomic sequence, every possible annotation of this nucleotide is represented in the graph. The graph is designed to model the whole prediction space: all consistent gene structures can be represented by a path through the graph, whose weight is defined as the sum of its edges weights. The minimum weight path defines the optimal prediction. Several sources of evidence are used to weight the edges of the graph and a shortest-path dynamic programming algorithm (linear in time and space) scans the graph to provide an optimal path which represents the best gene prediction according to available evidences.

#### Structure of the initial graph

**Figure 2:**
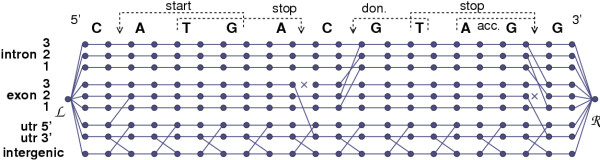
**EuGène's directed acyclic graph for a short example sequence**. For simplicity purposes, only the forward strand is considered. The DNA sequence is shown above the graph. Horizontal tracks represent the different possible annotations: intergenic (bottom), UTR 5' and 3', exon in the 3 frames, intron in 3 phases (the phase of an intron is defined according to the splicing position in the last codon of the previous exon). On each track, 2 vertices are used to represent each nucleotide. These 2 vertices are linked horizontally by a contents and a transition edge (see the text and Figure 4 for details). Dotted arrows show occurrences of biological signals (like start/stop codons and donor/acceptor splice sites). They produce additional transition edges at the corresponding position. Since this version of EuGÈNE does not include any promoter or polyA site prediction tool, transitions from intergenic to UTR and vice-versa are allowed at every nucleotide position. All consistent gene structures can be represented by a path connecting the initial and terminal vertices  and .

**Figure 3:**
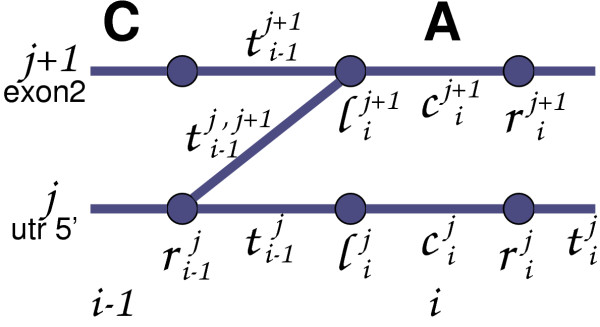
**Detail of EuGène's directed acyclic graph and algorithm**. The zoomed region contains the two first nucleotides of the example sequence of Figure 3 (C at position *i *- 1, and A at position *i*), and two annotation tracks (UTR5' for *j *and exon in frame 2 for *j *+ 1). The contents edges *c *connect the *l *vertices to the following *r *vertices of the same track. Transition edges *t *are either horizontal and link the *r *vertices to the *l *vertices of the same track, or transversal and link the *r *vertices to all possible *l *vertices according to the occurrence of a biological signal in the sequence. In this example, between  and  a vertex  allows the transition from the UTR5' track at position *i *- 1 to the exonic track at *i *because the A nucleotide at position *i *is the first nucleotide of a potential start codon ATG. The dynamic programming algorithm used in EuGÈNE determines, for each vertex *r*, which vertex precedes *r *in the optimal path. In this example, at position *i *for the track *j *the best path leading to  from the left has a weight  (only one origin is possible). For the track *j *+ 1, the best path leading to  will be attributed a weight of either , whatever the lower.

Each path through the graph represents a potential gene structure prediction for the genomic sequence (Figure 2). The graph is composed of *k *tracks that represent the possible annotations that can be attributed to each nucleotide (coding, intronic, intergenic and UTR, with specific strand and frame). Let ℓ be the genomic sequence length. For a given nucleotide's position *i *with (1 <*i *< ℓ) and for each track *j *with (1 <*j *<*k*) the two flanking vertices  and  are defined. Two edges are also built : a *contents *edge  linking  to  and a *transition *edge  linking  to  (Figure 3). Additional transition edges  are put from  to all  according to the occurrence of a potential biological signal allowing a switch from state *j *for the nucleotide at the position *i *to the state *j*' for the following nucleotide. For example, on the position *i *before the occurrence of an ATG, a transition edge  linking  to  (where *j *corresponds to the UTR5' track and *j*' is the coding exon track on the appropriate frame) is present, as illustrated in Figure 3. Two special vertices  and  are added at the extremities of the graph. They are respectively connected to all  and all . Initially, all edges are oriented from left (5') to right (3'). It is easy to see that all possible gene structure can be represented by a path from  to .

#### Weighting the graph

The weight of a path is the sum of all the weights of the edges in the path. The edges are weighted according to the evidences used. EuGÈNE can combine several sources of evidence such as probabilistic coding models, output of splice site or start codon prediction software and sequence similarities with transcripts, proteins, or other genomic sequences [33]. Contents and transition edges *c *and *t *are penalized respectively by weights *Wc *and *Wt *according to a weighting function characterized by parameters specifically set for the corresponding source of evidence. The set of parameters is optimized on a learning dataset by maximizing the overall accuracy of the software. For more information about the weighting methods, please refer to [22].

#### Example of transcript alignment integration

A transcript-to-genome alignment can easily be taken into account by weighting the appropriate edges of the graph. To favor a gene prediction in the alignment region, the intergenic track edges included in this region can be penalized by increasing their weight. More finely, the exon and the intron tracks edges can also be penalized at all positions involved respectively in a gap and in a match in the alignment. Thus, all gene structure prediction inconsistent with the transcript alignment information tends to be penalized. More drastically, it is possible to force the prediction to be consistent with the alignment by applying infinite penalty weights. Note that there are several such predictions since the start codon used is unknown and the transcript may be incomplete.

#### Initial algorithm

To identify the optimal path defined by the lowest weight, EuGÈNE uses a dynamic programming algorithm inspired from Bellman's shortest-path algorithm [34], also used for HMM in its Viterbi's version. Improvements of this algorithm allow EuGÈNE to take into account constraints on gene element lengths. For simplicity, we will not describe these sophistications in this paper. The algorithm of EuGÈNE associates to each vertex  a variable  which contains the weight of the optimal path from  to  and a variable  which contains the vertex that precedes  in this optimal path. The weight of this path can be computed recursively from 5' to 3' as:



A short example is displayed in Figure 3. The vertex  that minimizes this value provides the previous . At vertex , the best path is retrieved by a simple backtracing procedure through all *π*. This algorithm is linear in time and space in the length of the sequence (*O*(ℓ) complexity). It is important to note that the same algorithm can be used in a backward version (from  to ), by computing at each vertex  the weight  of the best path from  to  as .

### AS evidences integration

Given an *alternative transcript *genomic alignment, any prediction which is optimal among all the predictions that are consistent with the alignment evidence will be called an *alternative prediction*. Given the set of the previously detected *alternative transcripts*, we want EuGÈNE-M to produce a set of *alternative predictions *such that every *alternative transcript *has a corresponding prediction in this set. A simple way to produce such an alternative prediction would be to inject the exon-intron structure information given by the transcript alignment into the graph as described above (using infinite weights to force the prediction to strictly respect the alignment evidence), and then to execute EuGÈNE on the resulting graph. However, obtaining all *alternative predictions *would require one execution for each *alternative transcript*. *n *being the number of transcripts and *l *the genomic sequence length, this would result in a *O*(*ln*) time complexity, which is not appropriate for long genomic sequences and numerous transcripts.

Hopefully, this complexity can be drastically reduced. The general idea to achieve a realistic complexity is to duplicate the subsection of the graph region involved in an alignment to create a so called local "Parallel Graph Subunit" (PGS), connected to the main graph at its extremities. Each alignment information is taken into account as constraints in the corresponding PGS, in such a way that finding the optimal path going through the PGS provides a corresponding optimal alternative prediction.

#### Extending the graph model with PGS

**Figure 4:**
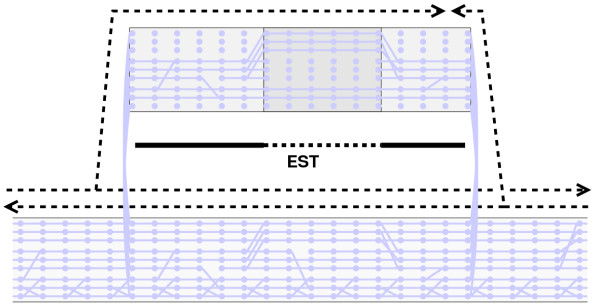
**Extension of EuGène's graph by a PCS to incorporate a single alternative transcript alignment**. From the main graph (bottom) described in Figure 3, a Parallel Graph Subunit (PGS) is built (above) by duplicating the whole graph section involved in the EST alignment (between the graphs). Gene structure evidences provided by the alignment are taken into account in the PGS by forbidding the intergenic track all along the alignment, intronic tracks at match positions (light grey), and exonic tracks in gap positions (dark grey). Dotted arrows represent the two algorithm scans, the forward version from left to right, and the backward version from right to left. At the junction point in the PGS, an optimal prediction is obtained. Figure not to scale.

For a transcript alignment that extends from position *g *to *h *on the genomic sequence, the entire subsection of the graph between *g *and *h *is duplicated to create a Parallel Graph Subunit (PGS) (Figure 4). This PGS is connected to the main graph at its extremities by special so-called *deviation *edges. For each track *j*, a deviation edge links the source vertex  in the main graph to its copy at the PGS left extremity, and another connects the source vertex  in the main graph to its copy at the PGS right extremity. The deviation edges are all oriented from the main graph to the PGS. The weights of the PGS edges, initially identical to the weight of the original edges, are modified according to the corresponding transcript alignment : gaps and matches forbid respectively the exonic and the intronic tracks, and the entire PGS intergenic track is forbidden.

#### Finding*alternative predictions*

**Figure 5:**
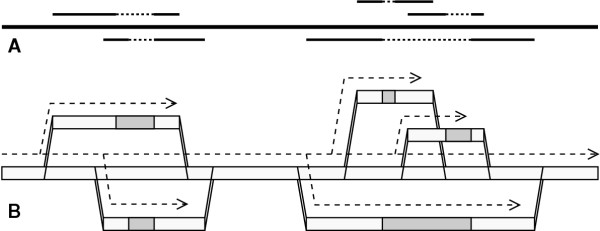
**Integration of several incompatible ESTs in EuGène-M's graph and algorithm**. A: EST alignments (plain lines represent exons, dotted lines, intron) on a genomic sequence (thick line). Each displayed EST is *incompatible *with at least another one. B: Multiple extensions of EuGÈNE's graph model after having processed these alignments. Each PGS (Figure 3) contains the information provided by its source EST. The dotted arrows show the algorithm progression through the resulting graph during the first scan, from the left to the right.

The modified algorithm proceeds in two steps. A first scan starts from  to  and applies the recursive formula described above to compute all , branching into each PGS (Figure 5). Thus, at each nucleotide's position and for each track (including those in the PGS), the weight of the optimal path from the left extremity  is identified. At , the optimal path is obtained by backtracing. Furthermore, for any given PGS, the cost of an optimal path going from , through the PGS and then to each of the righmost vertices  is known.

Then all edges (except the deviation) are reversed, and the backward version of the same shortest-path algorithm is used from  to  to compute all . This step ignores the PGS.

For a given PGS *A*, if we now consider the vertices  at the rightmost extremity of *A*, then the weight of an optimal path that goes from  to  through *A *can be computed as . From the given vertex, backtracing in both directions provides an optimal path that represents an optimal prediction in accordance with the transcript alignment evidence.

#### Output

Predictions are produced in the standard GFF format. The entire optimal annotation is first displayed, followed by the alternative ones. To enhance the readability and to avoid redundancy, for each *alternative prediction *the name of the corresponding transcript is mentioned and the region that differs from the optimal prediction is displayed. Besides, if several predictions are identical (regarding their predicted CDS only, UTR length differences being ignored), a single representative is displayed, along with the list of its associated transcripts.

### Computation time

The initial filtering and *incompatible *transcripts identification requires *O*(*n*^2^) pairwise comparisons. Each comparison is itself linear in the maximum number of introns in the transcript compared, which is typically bounded by a small constant and the whole process is therefore in *O*(*n*^2^).

The step that corresponds to the two dynamic programming scans (application of the recursive formula) requires a time and space complexity which is linear in the size of the input data. Indeed, if *L *is the total nucleic sequences length (genomic + kept *alternative transcript*), the weights of all (alternative and optimal) predictions can be computed in *O*(*L*).

For the backtracing and output step, since each alternative prediction has to be displayed in the region where it differs from the optimal one, and because this can extend beyond the alignment region, it is not possible to obtain an algorithm which is linear in the size of the input. However, it is possible to reach a linear complexity in the size of the output. This can be done by a simple modification of the standard backtracing procedure to avoid a full backtrace for each prediction. This is yet not implemented in the current version of the software.

A typical run of EuGÈNE-M on an AMD Athlon 1.7 GHz takes 47 sec. for a 500 kb BAC (for which 945 transcript alignments were kept after the first quality filtering step).

## Authors' contributions

SF designed and implemented the filtering algorithm as well as the double dynamic programming algorithm for AS prediction. He also ran the experiments on the Araset dataset. TS directed the research. All authors read and approved the final manuscript.

## Supplementary Material

Additional File 1 **EuGène's predictions on AraSet**. The additional file (SupplementaryFiles.tar.gz) contains gene structure predictions in the standard GFF text format for every AraSet sequence. ESTs detected as *Incompatible *by the method are displayed at the top of each prediction (if any). Click here for file

## Figures and Tables

**Table 1:** Analysis of the AS cases detected by EuGène-M in the AraSet genes data set. First, sequence IDs, genes and EST involved are reported. The TIGR and AtGDB columns indicate if each AS case is reported in these databases. The AS status is described as follows: ACC = alternative acceptor splice site, DON = alternative donor splice site, -EX = exon skipping (an entire exon lacks in the reported variant), +IN = intron inclusion (an internal part of an exon is spliced), FP = false positive AS. nt = nucleotide. Some ESTs of the At2g39780 gene in seq16 are not correctly aligned: the use of either GeneSeqer or sim4 with default options leads to a missed 4 nt exon (not involved in AS). In seq50, the 168 nt additional (+IN) intron from the EST CF652136 is flanked by GC-CT (instead of the canonical GT-AG). In seq62, the EST AV542276 from the gene At4g37040 overlaps with an intron of EST AV562725 from the neighboring gene At4g37050. In seq65, the EST BE521212 is not spliced between the exon 5 and 6 of the gene At2g44100 (intron retention case), and is thus suspected of incomplete maturation. Except for CF652136, all alignments can be browsed on the AtGDB site.

AraSet sequence	Gene ID	EST evidence	TIGR	AtGDB	AS status	Note
seq14	At2g47640	AI998209	Y	N	ACC	skip 3 nt
seq16	At2g39780	AV832175	N	N	-EX	incorrect alignments
seq50	At5g46290	CF652136	N	N	+IN	non consensus splice sites
seq53	At3g51800	AV544387	Y	Y	ACC	add 27 nt
seq62	At4g37070	AU236122	Y	Y	DON	add 33 nt
seq62	At4g37050	AV542276	N	N	FP	overlapping genes
seq65	At2g44100	BE521212	N	N	FP	incomplete splicing
seq65	At2g44120	BE524396	Y	N	ACC	skip 33 nt
seq69	At4gl4350	AV547538	Y	Y	+IN	skip 105 nt
